# *PgWRKY44*-mediated modulation of SA/JA pathways enhances blast pathogen resistance in pearl millet and rice

**DOI:** 10.1080/15592324.2026.2650899

**Published:** 2026-04-04

**Authors:** Baisista Saha, Sushmita Saha, Mufeeda KT, Jagatjeet Nayak, Jeky Chanwala, Nrisingha Dey, Mrunmay Kumar Giri

**Affiliations:** a School of Biotechnology, Kalinga Institute of Industrial Technology (KIIT) Deemed to be University, Bhubaneswar, Odisha, India; b Institute of Life Sciences, Bhubaneswar, Odisha, India; c Ramakrishna Mission Vivekananda Educational and Research Institute, Narendrapur Campus, Kolkata, West Bengal, India

**Keywords:** Pearl millet (*Pennisetum glaucum*), WRKY TF, *PgWRKY44*, biotic stress, transgenics, hormonal crosstalk, pathogen resistance

## Abstract

The complex network of plant defense mechanisms in response to various stress conditions is often tightly regulated by multiple transcription factors (TFs). They can alter their target gene expression upon binding to their respective cognate cis-elements. A number of studies have functionally characterized the role of different WRKY TFs in various stress conditions in plants such as *Arabidopsis*, rice, and tobacco. However, their role in a major C4 crop such as pearl millet remains elusive. In our previous study, we showed that the ectopic expression of the group IId member *PgWRKY44* in *Arabidopsis* provides enhanced tolerance to drought and saline conditions. However, along with *in silico* promoter analysis, the *GUS* reporter assays upon salicylic acid and methyl jasmonate application indicated its probable involvement in biotic stress resistance. Transcript profiling has shown enhanced expression of *PgWRKY44* during both pathogen infection and phytohormone treatments, suggesting its involvement in hormone-mediated defense pathways. To further investigate its role, we have generated transgenic lines overexpressing *PgWRKY44* in both pearl millet and rice, which showed elevated resistance against hemibiotrophic fungus *Magnaporthe grisea*, as evidenced by reduced lesion formation and significant changes in the expression levels of several defense-related genes. The transgenic lines exhibited elevated expression of key genes in both SA (e.g., *NH1*, *ICS1*, and *PAD4)* and JA (e.g., *PR10*, *LOX*, and *AOS2*) pathways, highlighting a potential role for *PgWRKY44* in modulating hormonal crosstalk in regulating defense response against *M. grisea* infection. Collectively, our findings establish *PgWRKY44* as a dual regulator of abiotic and biotic stress responses and demonstrate its utility in engineering disease-resistant, climate-resilient cereal crops.

## Introduction

Plants are easily affected by a number of environmental challenges, encompassing both abiotic and biotic stresses. Biotic stresses, which include infections caused by pathogenic fungi, bacteria, viruses, and nematodes, pose a major threat to agricultural productivity worldwide.^1^ To deal with these alarming threats, plants have evolved complex defense mechanisms that are precisely controlled across molecular, cellular, and physiological levels.^2^ At the core of these defense responses lies a network of signal transduction pathways, which involves a diverse array of transcription factors (TFs) that play a major role in controlling the expression of other defense-related genes.^3^

WRKY TFs have emerged as key regulators of plant immune responses owing to their roles in mediating both pathogen-specific and broad-spectrum resistance mechanisms. The differential expression patterns of these genes across various tissues and developmental stages, upon exposure to various abiotic and biotic stress conditions make them uniquely suitable to regulate plant defense mechanisms.^4^^,^^5^ WRKYs are recognized especially by a conserved DNA-binding domain (DBD) WRKY that identifies W-box elements with a signature sequence of [(T)TGAC(C/T)] in the promoter of their downstream target. Depending upon the number of DBDs present and structural differences of their zinc finger motifs, WRKYs are categorized in three major groups.^6^ Rapid activation of WRKY TFs has been reported against pathogen attack and is often involved in hormone signaling pathways connected with defense, particularly salicylic acid (SA) and jasmonic acid (JA) signaling.^7-9^ SA-mediated responses are commonly linked with systemic acquired resistance (SAR) and biotrophic pathogen defense, whereas JA signaling is considered to be connected with necrotrophic pathogens and herbivory resistance.^10^^,^^11^ WRKY proteins may function as positive or negative regulators in these pathways and typically act through complex feedback loops and cross-regulatory networks to modulate immune responses.^5^^,^^12^ Apart from their pivotal roles in finetuning plant immune responses against pathogens, several WRKYs exhibit their involvement in governing abiotic stress responses, where they orchestrate the gene regulatory networks to enhance tolerance against different environmental factors including drought, salinity, and temperature extremes.^13^^,^^14^ For example, improved tolerance against salinity, and water deficit condition has been reported in transgenic rice and *Arabidopsis* plants overexpressing *TaWRKY17,* and *TaWRKY24.*^15^^,^^16^ In rice seedlings significant enhancement in the heat and drought stress resistance was observed by the overexpression of *OsWRKY11*, regulated by the *HSP101* promoter.^17^ Transcriptome analysis under high and low temperature stress condition has revealed differential expression of several WRKY TFs from tomato and alfalfa plants.^18^^,^^19^ Several WRKY TFs from both monocot and dicot species are found at the junction of the plant response to this combined stress. GhWRKY25, a group I WRKY protein from *Gossypium hirsutum*, was identified to play a negative role in water deficit conditions and *Botrytis cinerea* infection, whereas it can act as a positive regulator during salt stress conditions in transgenic tobacco.^20^ Reduced tolerance to drought and the fungal pathogen *Rhizoctonia solani* was observed when another group III WRKY protein, GhWRKY27a, from *G. hirsutum*, was overexpressed in tobacco plants. In contrast, silencing of the same in cotton caused heightened resistance in water-deficient conditions.^21^ Taken together, these findings suggest the involvement of WRKY TFs as one of the major players in regulating the tolerance mechanisms against both biotic and environmental stress.

Although extensive characterization of several WRKY proteins from in model plants like *Arabidopsis* and *Oryza sativa* has been done, functionally much less is known about them in climate-resilient cereal crops like pearl millet (*Pennisetum glaucum*).^22-24^ Pearl millet is a nutritionally enriched C4 cereal crop, which shows strong adaptability to arid and semi-arid climatic conditions, and holds significant promise for sustainable agriculture under changing climate conditions.^25^^,^^26^ The availability of genome sequence, published by Varshney et al.^27^ and Yan et al.^28^ provides priceless insights into genome composition and architecture, helping to bridge existing research gaps.^27^^,^^28^ Genome wide analysis performed by our group recently has revealed the presence of 97 putative WRKY transcription factors of pearl millet.^29^ In our previous study, a Group IId WRKY TF, *PgWRKY44* from pearl millet was identified, which is localized inside the nucleus with no self-transcription activation ability.^22^ The functional characterization of this gene has demonstrated its role in conferring tolerance to multiple abiotic stresses, including salinity, drought, and heat. Ectopic expression of *PgWRKY44* in *Arabidopsis* enhanced stress tolerance by modulating ABA-mediated signaling, improving ROS scavenging, and activating a range of stress-responsive genes. Additional analysis of the *PgWRKY44* promoter identified several biotic stress-associated cis-regulatory elements (CREs), such as MeJA-responsive motifs (TGACG-motif), SA-responsive elements (as-1), MYB binding sites (MBS), W-box, STRE, and wound-responsive elements (WUN-motif). Together, these results imply that *PgWRKY44* might play a role in biotic stress responses in plants.

In our present research, we planned to investigate *PgWRKY44'*s functional roles in the disease defense mechanism, specifically its response to infection by *Magnaporthe grisea* (syn. *Pyricularia oryzae* and *P. grisea*). Transgenic pearl millet and rice plants constitutively expressing *PgWRKY44* were generated, and their response to pathogen infection was analyzed. We also conducted promoter activity assays and expression profiling of key genes involved in defense associated with SA- and JA-dependent pathways to dissect the transcriptional regulatory function of *PgWRKY44* under biotic stress conditions. The findings of this study highlight the potential of *PgWRKY44* as a target for a crop improvement program to develop climate-smart, pathogen-resistant crops that would help to advance sustainable agriculture.

## Materials and methods

### Plant materials, growth conditions, and stress treatment

*P. glaucum* (cv. 7042S) and *O. sativa* (cv. IR64) seeds were obtained from the International Crops Research Institute for the Semi-Arid Tropics (ICRISAT), Patancheru, Hyderabad, and ICAR-NRRI Cuttack, Odisha, respectively. The seeds were grown in a soil mix of equal ratios of black and red soils in a glasshouse under ambient growth conditions (25 °C ± 2 °C, 16-h photoperiod and 8-h dark period). For expression profiling of *PgWRKY44* in different tissues, leaves, stems, flag leaves, inflorescences, and seed material were harvested. For the hormone treatments, the leaves of four-week-old plants were sprayed with 100 μM salicylic acid (SA) and methyl jasmonate (MeJA) ^30^ prepared in autoclaved distilled water and 0.1% Tween 20. As leaves are the primary sites of photosynthesis, metabolism, and pathogen interaction, makes them an effective target tissues for studying hormone-induced defense responses. The rapid absorption of signaling molecules through the stomata and cuticular pathways allows phytohormones to quickly reach mesophyll cells and activate downstream signaling cascades. This method closely mimics natural defense signaling.^31-34^ For pathogen treatment, *M. grisea* pure culture MTCC1477 was procured from the Microbial Type Culture Collection and Gene Bank (MTCC), Chandigarh, India, and periodically subcultured on potato dextrose agar (PDA) media plate. 14-d-old seedlings were sprayed with *M. grisea* conidial suspension, adjusted to 4 × 10^5^ ml^−1^ dissolved in distilled water with 0.1% Tween 20. Plants treated with only water and 0.1% Tween 20 were taken as experimental controls. All the experiments were performed at least twice. For RNA isolation, samples were directly collected in liquid nitrogen and kept in a −80 °C freezer till processed.

### Promoter activity assay during stress conditions

*In silico* analysis of *PgWRKY44* 1000 bp upstream region was performed using online tools such as PlantCARE, NewPLACE, and PlantPAN 4.0.^35-37^ To check the transcriptional ability, the 1000 bp *PgWRKY44* promoter construct with the *GUS* reporter gene^22^ (kindly provided by Dr. N. Dey, ILS Bhubaneswar) was used for transient expression of the reporter *GUS* gene. Rice calli were transformed with the linearized double-stranded vector control and promoter construct using the peptide-mediated transformation method. After 2 d of co-cultivation, transformed calli were transferred to different callus induction media containing either 100 μM of SA, or 100 μM of MeJA. Additionally, to check the promoter activity after pathogen infection, the transformed calli were also placed on callus induction media after dipping in *M. grisea* spore suspension (4 × 10^5^ spores per ml) for five minutes, followed by drying on sterile filter paper. Both the hormone and *M. grisea-*treated calli were then kept in the dark for two days. For histochemical staining, samples were immersed in a solution containing 1 mM X-Gluc, 50 mM phosphate buffer (pH 7.0), 1 mM each of potassium ferrocyanide and ferricyanide, 10 mM EDTA, and 0.1% (v/v) Triton X-100, followed by overnight incubation at 37 °C.

### Construction of the plant overexpression vector and transgenic development

The mature transcript of *PgWRKY44* (1098 bp) was PCR amplified from the previously cloned *PgWRKY44*-pGEM-T easy construct as a template^22^ and cloned downstream to the maize ubiquitin promoter in the modified pCAMBIA1300 plant binary expression vector via *BamH*I and *Sma*I sites. Transgenic pearl millet plants were developed by the peptide-mediated transformation method with Reagent A of the VisTrans Plant Transfection Kit according to the manufacturer's protocol (VisTrans Plant Transfection Reagent, Cat No: VAS20191). In brief, the T-DNA region carrying the gene of interest (GOI) and the hygromycin selection cassette was PCR amplified using border-specific primers with high-fidelity DNA polymerase from New England Biolab (Cat no: M0530S), and after purification, it was used for the transformation of three-day-old seedlings of pearl millet. *Agrobacterium-mediated* transformation was performed to develop transgenic rice plants; for that, the callus of the rice cv. IR64 was infected with *Agrobacterium tumifaciens* (LBA4404) cells carrying the *PgWRKY44* construct, and putative transgenics were selected using hygromycin antibiotic selection. Seeds harvested from both putative transgenics (*T*_0_) were placed on half MS + 50 mg/L hygromycin media plates. Further confirmation of positive plants was done by PCR screening with *HptII* sequence-specific primer, and the process continued upto the third generation. All future experiments were conducted using plants obtained from T_3_ homozygous lines.

### Isolation of total RNA and qRT-PCR analysis

RNA isolation was done from different tissues of pearl millet and rice plants using RNAisoplus reagent (Takara Bio Cat. #9108) following a standardized protocol. First-strand cDNA was synthesized using the Verso cDNA Synthesis Kit (Thermo Scientific™-AB1453A) following the confirmation of RNA integrity via denaturing agarose gel electrophoresis. The RNA concentration was determined using spectrophotometric analysis. Expression profiling was done using QuantStudio™ 5 Real-Time PCR System (Applied Biosystems, Foster City, CA, USA). A reaction mixture of 20.0 µl was prepared containing 10.0 µl of PowerUp™ SYBR™ Green Master Mix (Applied Biosystems™-A25742), 0.4 µl of each forward and reverse primer (10 μM), and 4 µl (10 ng) of cDNA, and the volume was adjusted with nuclease-free water. NTC (non-template control) mixtures were also taken, having all the elements except the cDNA template. The amplification parameters were set as follows: initial denaturation at 95 °C for 10 min, followed by 40 cycles of denaturation at 95 °C for 15 s and 58 °C for 1 min. Reactions were setup in two technical and three biological replicates. For data normalization purpose, *GAPDH* and *Actin* were used as endogenous controls. The primers used in the studies are included in Supplementary Table S1.

### Pathogen spot inoculation assay

A spot inoculation assay was performed to analyze the effect of *PgWRKY44* overexpression in pearl millet and rice plants as described by Jia et. al. (2023) with slight changes.^38^ A detached uniform leaf at the 4thaf stage from wild-type and transgenic plants grown in a greenhouse was used for the assay. Around 5 cm leaf segments were cut into pieces and placed on petri plates with moist filter paper. Three 5-μl droplets each containing a conidial suspension of *M. grisea* with 0.02% Tween 20 (v/v) and 0.2% gelatin (w/v). The Petri dishes were kept at room temperature with fluorescent light and photographed 4 d after inoculation.

### Expression profiling of antioxidant and stress-related marker genes

The relative expression level was measured for major antioxidant genes from rice, including *CAT (catalase), POD (peroxidase),* and *SOD (superoxide dismutase),* and their putative homologs in pearl millet were analyzed under mock and pathogen-treated conditions using quantitative real-time PCR (qRT-PCR). Transcript levels of two PR genes (*PR1α* and *PR10*) were also analyzed in both the transgenic and wild-type pearl millet and rice samples following treatment with either the pathogen or a mock. To further elucidate the defense signaling pathway influenced by *PgWRKY44,* the expression levels of several stress-responsive marker genes linked with salicylic SA and JA-mediated defense signaling pathways—namely, *Isochorismate Synthase 1 (ICS1), Phenylalanine Ammonia-Lyase 1 (PAL1), Phytoalexin-Deficient 4 (PAD4), Arabidopsis NPR1 Homolog 1 (NH1), Lipoxygenase (LOX), Allene Oxide Synthase 2 (AOS2),*—were analyzed in rice samples using qRT-PCR.

### Statistical analysis

All experiments were run at least twice. A Student's *t*-test between mean values was employed to identify significant difference level after the mean and standard deviation of three biological replicates (*n* = 3) were determined. (***p < 0.001; **p < 0.01; and *p < 0.05).

## Results

### Transcripts of *PgWRKY44* expressed in different tissues of the plants

**Figure 1.**
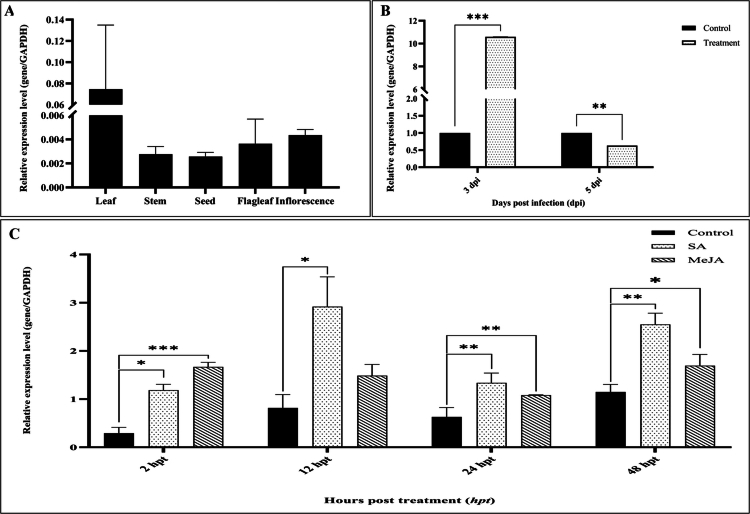
Expression analysis of *PgWRKY44.* (A) Transcript levels of *PgWRKY44* in different tissues. (B) Expression pattern of *PgWRKY44* upon treatment with the fungal pathogen *M. grisea*; (C) Expression profiling after phytohormone treatment (100 μM SA and 100 μM MeJA). Data of the three transgenic lines are presented as the means (±SDs) of duplicates. Asterisks indicate the level of significance determined by one-way ANOVA compared to corresponding untreated samples (***p < 0.001; **p < 0.01; and *p < 0.05).

Expression profiling of *PgWRKY44* in different tissues of pearl millet plants was done using qRT-PCR, including leaf, stem, seed, flag leaf, and inflorescence. *PgWRKY44* has shown its expression in all parts of the plant, with the maximum expression in the leaf tissue, followed by the inflorescence and flag leaf. In contrast, the expression was relatively low in the stem and lowest in the seed (Figure 1A).

### *PgWRKY44* expresses differentially in response to pathogen and phytohormone treatment

Involvement of *PgWRKY44* in the stress response against the fungal pathogen *M. grisea* was identified. As shown in Figure 1B, the accumulation of the *PgWRKY44* transcript was induced significantly by about 10-fold compared to the control after 3 d post-treatment, followed by a decrease after 5 d. In this study, the possible involvement of *PgWRKY44* in the phytohormone-mediated defense signaling pathway was analyzed in wild-type pearl millet cv. 7042S plants. Foliar spray of SA and MeJA has elevated the expression of *PgWRKY44.* Specifically, significant upregulation after 2, 12, 24, and 48 h for both SA and MeJA treatment (Figure 1C) has been observed.

### Characterization of *PgWRKY44* promoter in stress conditions

**Figure 2.**
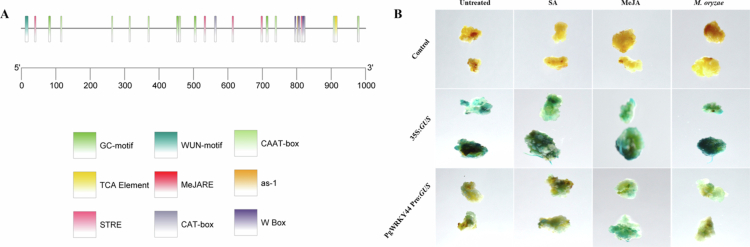
Characterization of *PgWRKY44* Promoter region. (A) *In silico* analysis of *PgWRKY44* promoter showing different CREs like GC-motif, WUN motif, MeJA responsive elements, and SA responsive elements (as1) etc.; (B) Histochemical *GUS* assay of transformed calli expressing the *GUS* construct in rice calli. Representative images showing *GUS* (β-glucuronidase) expression in putative transformed rice calli after peptide-mediated transformation with binary vectors harboring the *GUS* reporter gene. The top panel shows non-transformed control calli; the middle panel displays transformed calli with the CaMV35S promoter construct subjected to GUS staining; the lower panel shows the transgenic calli with the *PgWRKY44* promoter construct subjected to GUS staining. Blue staining confirms successful transgene expression in the transformed tissues. Each column represents an independent event or line.

The *in silico* study of 1000 bp upstream region of *PgWRKY44* has shown the occurrence of several cis-regulatory elements (CREs) majorly involved in the biotic stress response in the promoter region (Figure 2A). such as CCAAT box, WUN- motif, W-box, TCA-element, as1 elements, MeJA responsive elements etc. This indicates the probable involvement of *PgWRKY44* in phytohormone-mediated biotic stress signaling pathways. To further validate the *in silico* prediction of the transcriptional ability of the 1000 bp upstream *PgWRKY44* promoter was checked in 2-week-old rice callus. The intensity of *GUS* histochemical staining of the transiently transformed rice calli confirmed that the ability of the promoter to drive the *GUS* reporter gene was higher in the samples kept with 100 μM MeJA, followed by 100 μM SA and the fungal pathogen *M. grisea* treatment, as compared to the control samples. The coloration in samples carrying the CaMV35S promoter, which drives constitutive expression of the *GUS* reporter gene, was relatively uniform across all the treatments (Figure 2B).

### Constitutive expression of *PgWRKY44* in pearl millet and rice provides resistance against fungal pathogen *M. grisea*

**Figure 3.**
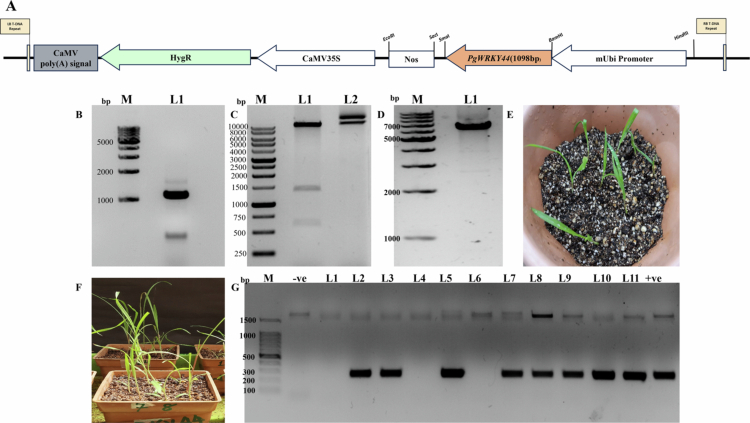
*PgWRKY44* overexpression vector construction and development of transgenic pearl millet plants. (A) Schematic diagram of pCambia1300_mUbi_*PgWRKY44*_NosT construct map; (B) PCR amplification of *PgWRKY44* (1098 bp) coding sequence; (C) Clone confirmation of pCambia1300_*PgWRKY44* overexpression construct via digestion with *BamH*I and *EcoR*I restriction enzymes, expected fragments – 10311 bp, 1385 bp, and 613 bp (L1), Undigested plasmid (L2); (D) Purified PCR product of *PgWRKY44* overexpression cassette along with *HptII* selection marker from T-DNA region (6577 bp); (E) 3 d old seedlings transferred to soilrite after transformation with Reagent A of VisTrans plant transfection kit; (F) 4th leaf stage plants selected for PCR screening; and (G) Agarose gel electrophoresis of PCR products amplified with gene specific forward primer and Nos terminator specific reverse primer (244 bp) to identify putative transgenics.

**Figure 4.**
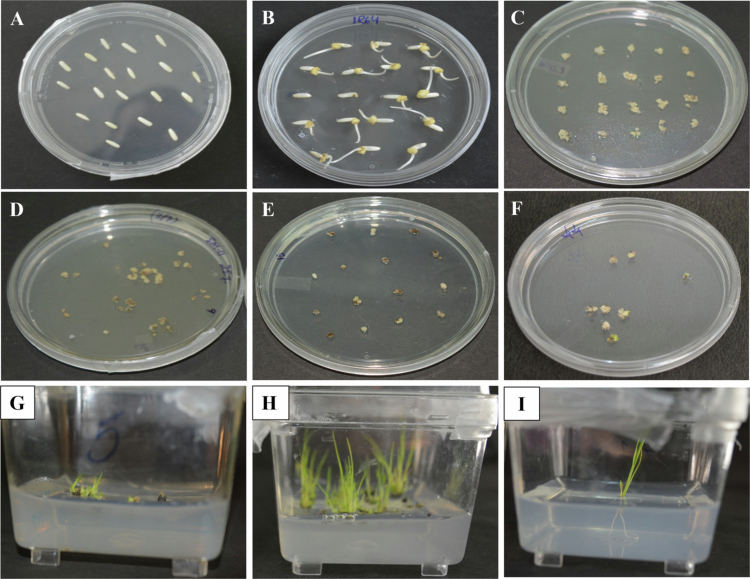
Development of *PgWRKY44* overexpressing lines of Rice through *Agrobacterium-*mediated transformation. (A and B) Rice seeds germination and Callus induction, (C) Callus co-cultivation after transformation; (D–F) *PgWRKY44* transformed calli on selection media; (G and H) *PgWRKY44* transgenics on shooting media; and (I) *PgWRKY44* transgenics on rooting media.

Differential expression of *PgWRKY44* transcripts and its promoter activity during pathogen and phytohormone treatments indicates the likely involvement of this WRKY gene against biotic stress. To confirm this, we developed T_3_ homozygous lines of pearl millet cv 7042S (Figure 3) and rice cv. IR64 (Figure 4), which constitutively express *PgWRKY44* driven by the maize ubiquitin 1 (Ubi-1) promoter.

**Figure 5.**
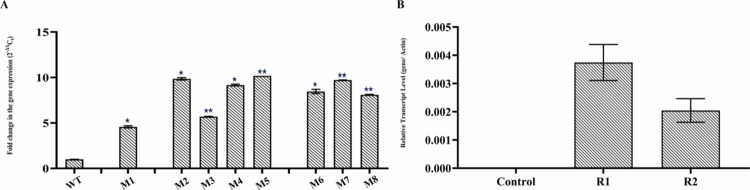
Expression analysis of *PgWRKY44* in T₃ transgenic lines of pearl millet and rice. (A) Pearl millet lines: M1 (Line 1), M2–5 (Line 2), and M6–8 (Line 3) and (B) rice lines: R1 (Line 1) and R2 (Line 2). Data of the three transgenic lines are presented as means (±SDs) of duplicates. Asterisks indicate the level of significance determined by one-way ANOVA compared to corresponding untreated samples (***p < 0.001; **p < 0.01; and *p < 0.05).

**Figure 6.**
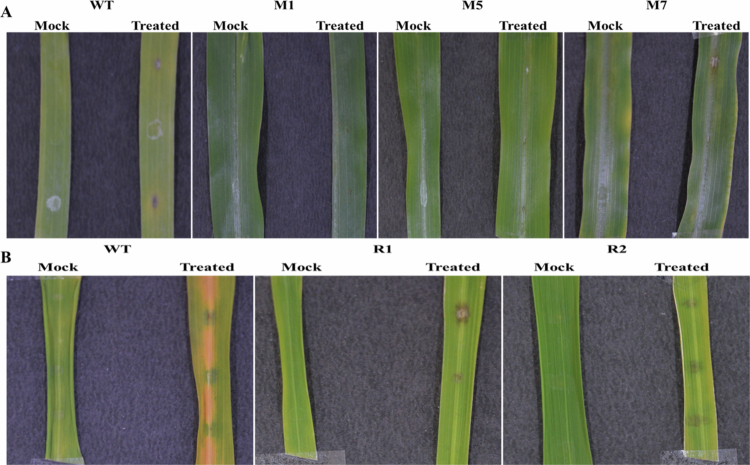
Evaluation of disease resistance through pathogen spot inoculation assay. (A) Representative leaf images of T_3_ homozygous transgenic lines of pearl millet and (B) rice and their respective wild-type (WT) plants after spot inoculation with mock and *Magnaporthe grisea*.

We obtained three independent lines of pearl millet and two independent lines of rice and confirmed them by PCR screening with *HptII* specific primer (Supplementary Figure S1). They show no morphological differences compared to their wild types. Three T_3_ homozygous independent lines showing highest expression from pearl millet (Figure 5A) and two rice plants (Figure 5B) were selected for the pathogen spot inoculation assay. After four days, we observed that more severe lesions formed around the inoculated spots on the wild-type leaflets, as compared to their transgenics (Figure 6).

### Expression profiling of *CAT*, *POD*, and *SOD* antioxidant genes during pathogenesis

**Figure 7.**
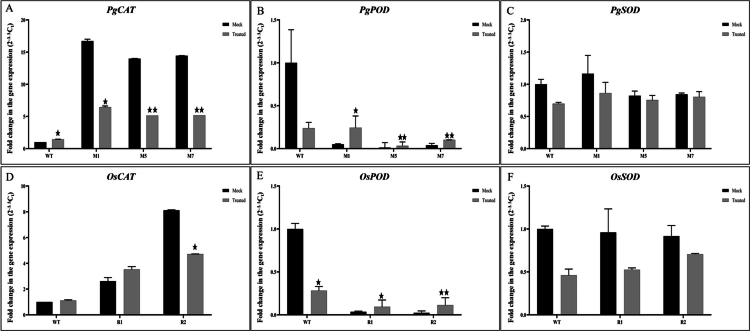
Relative expression profiling of antioxidant genes upon pathogen challenge (*M. grisea*). Relative transcript levels of the antioxidant genes CAT, POD, and SOD were analyzed in the wild-type and transgenic lines following pathogen challenge. (A–C) Expression profiles of *PgCAT*, *PgPOD*, and *PgSOD* in pearl millet lines (WT, M1, M5, and M7); (D–F) Expression profiles of *OsCAT*, *OsPOD*, and *OsSOD* in rice lines (WT, R1, and R2). Data of the three transgenic lines are presented as means (±SDs) of duplicates. Asterisks show significant difference determined using one-way ANOVA compared to control (***p < 0.001; **p < 0.01; and *p < 0.05).

The basal level expression of *CAT* was found to be significantly induced in the transgenic lines of both rice (Figure 7D) and millet (Figure 7A) as compared to their wild type one in mock treated samples. Although during pathogen infection, the expression level was observed to be reduced or somewhat unchanged. A significant reduction was observed for the expression of *POD* in both the rice (Figure 7E) and millet (Figure 7B) transgenics in comparison with their wild type, but unlike the wild type the expression level was induced after pathogen treatment as compared to the mock-treated samples. However, the overall expression of *SOD* remained mostly unaffected in all the samples (Figure 7C and F).

### Expression profiling of genes involved in the defense mechanism modulated by *PgWRKY44*

**Figure 8.**
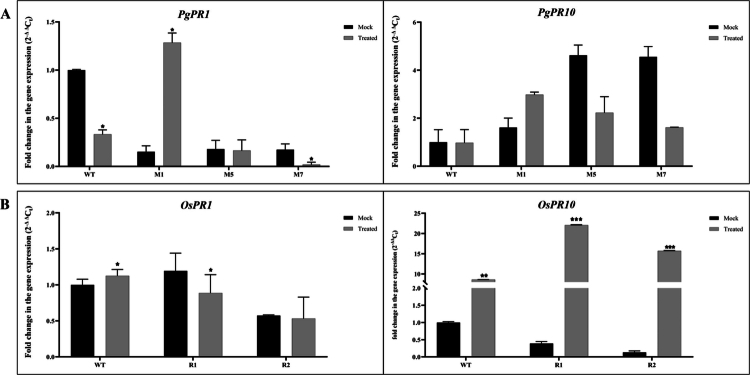
Relative expression profiling of pathogenesis-related (*PR)* genes upon pathogen challenge (*M. grisea*). Transcript levels of *PR* genes from(A) pearl millet and (B) rice; gene expression levels in transgenic plants were quantified relative to those in non-inoculated wild-type plants. Data of the three transgenic lines are presented as means (±SDs) of duplicates. Asterisks show significant difference determined using one-way ANOVA compared to control (***p < 0.001; **p < 0.01; and *p < 0.05).

Transcript profiling of two pathogenesis-related (*PR*) genes and six other genes recognized for their role in the SA- and JA-mediated defense signaling pathway was examined in the constitutively expressed *PgWRKY44* transgenic lines. Pathogen challenge has induced the transcript accumulation level of the *PR10* gene remarkably in rice plants (Figure 8B), whereas the expression level of *PR1α* seems to be most likely unaffected by pathogen challenge.

**Figure 9.**
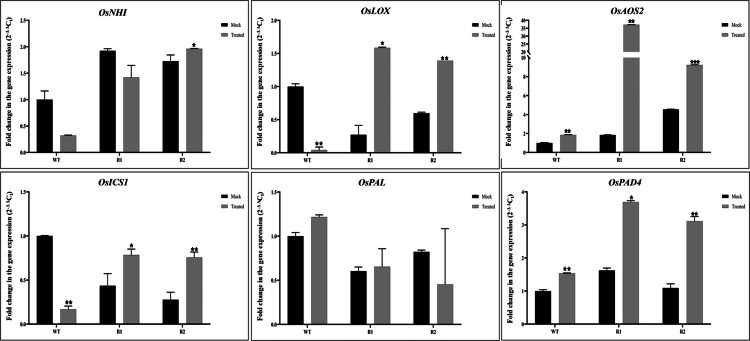
Transcript levels of marker genes from the SA and JA biosynthesis pathway from rice. Gene expression levels in transgenic plants were quantified relative to those in non-inoculated wild-type plants. Data of the three transgenic lines are presented as means (±SDs) of duplicates. Asterisks show significant difference determined using one-way ANOVA compared to control (***p < 0.001; **p < 0.01; and *p < 0.05).

Further analysis of the genes like *NH1, ICS1, and PAD4,* which are involved in SA-mediated defense signaling, were found to be significantly induced after pathogen challenge in transgenic rice plants. However, the expression patterns of *PAL* were noticed to be unaffected by pathogen infection, with lower expression in the transgenic plants compared to wild-type ones. Moreover, the upregulation of JA biosynthetic genes like *LOX* and *AOS2* indicated the JA-mediated disease resistance mechanism in *PgWRKY44* transgenic rice plants (Figure 9).

## Discussion

Pearl millet is one of the major C4 family crops cultivated in the semi-arid region of Southeast Asia and Africa, including India, after rice, wheat, and sorghum.^39^ In comparison to other cereals such as rice, maize, and sorghum, pearl millet can easily tolerate adverse environmental conditions such as higher temperatures and low moisture in the soil. But the disease susceptibility of pearl millet towards several pathogens, such as *M. grisea, Sclerospora graminicola,* and *Claviceps fusiformis,* is still a major concern, as it limits the growth and yield of the plant.^40-42^ Plants have their innate ability to withstand these adverse conditions. Upon pathogen attack, the defense signaling mechanism of plants gets activated through numerous signaling molecules and the fine-tuning of several genes or TFs that play a crucial role in defense. WRKYs play an exclusive role in plant defense responses to environmental stresses through hormone-mediated signaling pathways. Several WRKY proteins have been reported to be involved in disease resistance in dicotyledonous and monocotyledonous plants.^43^^,^^44^ In pearl millet, the functional characterization of WRKY TFs has been very recently done in response to salt and drought stress.^22-24^ However, the molecular function and mechanism against biotic stress remained untouched. Our findings in this study reinforce the importance of WRKY TFs in dual stress responsiveness, as *PgWRKY44* not only contributes to abiotic stress tolerance, as reported previously,^22^ but also plays a significant role in resistance against the fungal pathogen *M. grisea.*

To explore the involvement of *PgWRKY44* in response to biotic stress, its expression profile was examined after pathogen challenge. Our findings confirmed that the *PgWRKY44* transcript level was significantly induced. Apart from that, the exogenous application of phytohormones like SA and MeJA also modulated the transcript level of *PgWRKY44*, as phytohormones are important signaling molecules involved in a plant's defense mechanism. This suggests that *PgWRKY44* may be involved in both JA- and SA-mediated defense signaling pathways, which is further supported by the existence of CREs like W-box, TGACG-motif (MeJA-responsive), and as-1 (SA-responsive) in its promoter region.^22^ Notably, the GUS histochemical staining assays also confirmed the enhanced transcriptional ability of the *PgWRKY44* promoter under hormone and pathogen treatments. These observations together indicate that *PgWRKY44* can act in positive regulation of the biotic stress response mechanism via the modulation of the hormone-mediated defense signaling pathway.

Transgenic lines of rice and pearl millet overexpressing *PgWRKY44* were generated to further understand its functional role. These plants have shown enhanced resistance towards *M. grisea* pathogen stress treatment, without any significant growth penalties, suggesting that *PgWRKY44* could be useful for crop improvement.

In rice, four members of the PR10 family have been characterized so far, namely, *PR10a*, *PR10b*, *JIOsPR10* (jasmonic acid inducible PR10), and *RSOsPR10.*^45-47^ These PR10 genes are reported to be induced by *Magnaporthe oryzae* infection and jasmonic acid treatment, suggesting their involvement in plant defense responses and possible functional redundancy. In coherence with this both *PR10* and *NH1,* the key genes of JA and SA pathways, respectively, were upregulated upon pathogen exposure in the *PgWRKY44* rice transgenic lines, suggesting simultaneous activation of both pathways, aligned with the findings of Chern et. al. 2005, where they have observed that overexpression of *NH1* can enhance the expression of rice *PR10.*^48^ Interestingly, the expression of *PR1α,* a marker gene for the SA pathway, was found to be inhibited upon pathogen infection, possibly due to the antagonistic effect of the JA pathway.

Some of the key enzymes of JA biosynthesis, like *LOX* and *AOS2,* showed enhanced expression in transgenic rice plants upon pathogen exposure, suggesting that *PgWRKY44* may help to coordinate a JA-mediated disease resistance against *M. grisea,* probably by suppressing the SA signaling pathway downstream of SA accumulation. This finding is in line with earlier reports in rice and *Arabidopsis*, where WRKY members like *OsWRKY45, AtWRKY33,* and *AtWRKY70* have shown differential responses by modulating hormonal crosstalk.^49-51^

**Figure 10.**
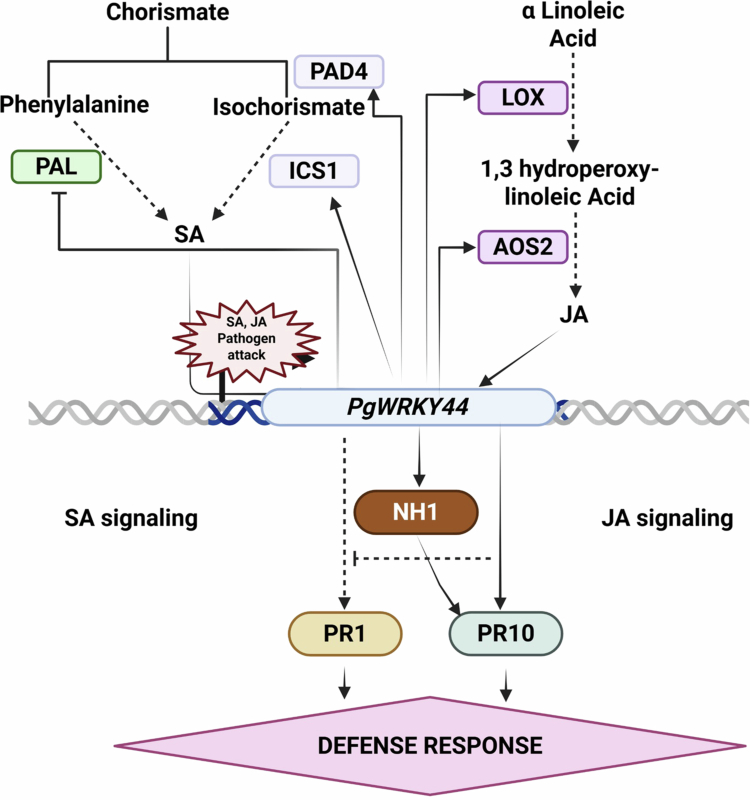
A proposed model illustrating the role of *PgWRKY44* in salicylic acid (SA)- and jasmonic acid (JA)-mediated defense signaling pathways in pearl millet. Arrow-headed lines indicate activation, and lines ended with perpendicular small lines show inhibition.

As demonstrated, *PgWRKY44* overexpression was affected by both JA and SA (Figure 1), and the constitutive expression of *PgWRKY44* changes the expression levels of *PR* genes and *NH1* (Figure 8); it can be assumed that one action site for *PgWRKY44* might be downstream of SA and JA and upstream of *NH1* and *PR* genes (Figure 10). Both SA and JA can induce the expression of *PR* genes, but in our study, the expression pattern clearly shows that *PgWRKY44* can induce the *PR10* gene during pathogen attack while suppressing the expression of the *PR1* gene overall. The pathogen-induced activation of the *PR10* gene observed in this study is consistent with previous reports by Kim et al. (2003) and Kim et al. (2008), which demonstrated that the expression of rice PR10 (JIOsPR10) is induced by rice blast fungus infection as well as by jasmonic acid treatment.^52^^,^^53^ The expression patterns of *ICS1* and *PAD4* was found to be opposite to those of *PR1,* suggesting that the activation of the SA pathway through the ICS pathway. Several studies have demonstrated that an increase in *ICS1* and *PAD4* expression promotes SA biosynthesis and amplifies the defense mechanism. *ICS1* is a key enzyme that converts chorismate to isochorismate, a major step for SA biosynthesis during pathogenesis.^54^^,^^55^ *PAD4* acts as a positive regulator working upstream to enhance the expression of *ICS1* and SA accumulation, functioning together along with *EDS1* in a positive feedback loop.^56^^,^^57^ Collectively, our results indicate that *PgWRKY44* may help to activate SA biosynthesis through the isochorismate pathway, while decreased *PR1* expression suggests that downstream signaling may be disrupted or negatively regulated to promote the defense response predominantly via the JA-dependent pathway.

Pathogen invasion typically triggers a rapid reactive oxygen species (ROS) burst, which functions as an early signaling event in plant immunity.^58^ However, excessive accumulation of ROS may lead to oxidative damage to the cellular components. Therefore, plants maintain a delicate balance between ROS production and detoxification through antioxidant enzymes.^59^^,^^60^ In addition to the identification of phytohormone-mediated stress signaling pathways, we have evaluated the distinct modulation of some major antioxidant enzyme genes, such as *CAT (catalase), POD (peroxidase),* and *SOD (superoxide dismutase),* under mock and blast-treated conditions, highlighting WRKY's role in fine-tuning reactive oxygen species (ROS) homeostasis during pathogen challenge. The activity of *CAT* converts H_2_O_2_ into oxygen and water to maintain the balance of ROS in the cell.^61^^,^^62^ In the current study, the *CAT* activity was significantly increased, suggesting that WRKY-mediated blast resistance may involve efficient detoxification of pathogen-induced ROS by catalase. This finding can be supported by the finding of Yasmin et al., 2023, where the increased activity of CAT was observed in blast resistant variety BAUdhan 3 when compared with susceptible rice variety BRRIdhan 28.^63^ In contrast, the basal level expression of *POD* in both the rice and pearl millet transgenics was reduced relatively in the transgenic rice lines compared with the wild type. However, after pathogen infection there was a significant increase in *POD* expression was observed only in the transgenic plants. Taken together the result suggests that the higher expression of *CAT* in the transgenic lines as compared to their wild types and that *POD* expression after pathogen challenge in the transgenic plants might contribute to the disease resistance phenotype of both the rice and pearl millet transgenic plants.

Besides this, our previous data from *PgWRKY44* network analysis with tobacco and *Arabidopsis* indicated the involvement of different calmodulin family members, such as CAM3 and 5 from *Arabidopsis* and CAM3, 7, and 12 from tobacco, in salinity and drought stress. Furthermore, previous studies have shown the role of different calmodulin and calmodulin-like proteins in response to biotic stress.^64^^,^^65^ A detailed analysis of these calmodulin family proteins in *PgWRKY44* transgenic plants may further elucidate the underlying mechanisms of the disease response.

Using the STRING online server (https://string-db.org), we also identified several potential *PgWRKY44*-interacting partners implicated in biotic stress responses, such as mitogen-activated protein kinases (MPKs), sigma factor-binding proteins (SIBs), G-box-binding factors, and camalexin synthase, etc.^62^^,^^66-70^, which might have some implications in regulating biotic stress responses through *PgWRKY44*. Homologs of these putative targets were identified in the pearl millet genome (supplementary table S2). Studies have shown that interactions between WRKY transcription factors and MAP kinases in rice play critical roles in resistance to various pathogens, including the blast fungus Magnaporthe oryzae.^71-73^ Downregulation of MPK3 and MPK6 enhances blast resistance by promoting jasmonate signaling and diterpenoid phytoalexin biosynthesis.^74^^,^^75^ In this study, we quantified the transcript levels of two MAP kinases, OsMPK3 and OsMPK6, in rice and their putative homologs in pearl millet before and after pathogen inoculation (Supplementary Figure S2). No significant pathogen‑induced changes in MPK expression were detected in either the wild‑type or *PgWRKY44*‑overexpressing lines. However, both rice MPKs and their pearl millet homologs showed significantly reduced basal expression in the transgenic lines compared with their corresponding wild types. These data suggest that constitutive overexpression of *PgWRKY44* may transcriptionally repress MPK3/MPK6-type kinases, thereby contributing to enhanced disease resistance in the transgenic lines. These findings suggest that *PgWRKY44* overexpression transcriptionally represses MPK3/MPK6-type kinases, thereby boosting resistance—consistent with the findings of Hu et al. (2015), where OsWRKY53 overexpression reduces *OsMPK3/OsMPK6* transcripts via direct physical interaction, while silencing increases their expression.^72^ Further functional characterization of these candidate genes is necessary to gain a comprehensive understanding of the signal transduction pathways regulated by *PgWRKY44* *in vivo* during pathogenesis.

## Conclusion

In summary, an in-depth analysis of a Group IId WRKY transcription factor from pearl millet, *PgWRKY44*, and its 1,000 bp upstream promoter region demonstrates its essential role in stress tolerance against the fungal pathogen *M. grisea.* Through overexpression of *PgWRKY44* in rice and pearl millet, we highlighted that *PgWRKY44* enhances disease resistance by modulating key genes that play important role in the SA and JA signaling pathways. Additionally, promoter analysis and expression profiling under different stress conditions support the inducibility and regulatory potential of *PgWRKY44* during biotic stress. While we have checked the transcript levels of genes involved in hormone signaling pathways in transgenic rice plants, further investigation of transgenic pearl millet is necessary to determine whether a similar defense signaling pattern is followed across the species or not. Promoter engineering and targeted gene editing utilizing the potential of the CRISPR-Cas system could exploit *PgWRKY44's* regulatory potential to tailor plant defense responses in a more controlled manner. Furthermore, the dual functionality of *PgWRKY44* in both abiotic and biotic stress tolerance underscores its value as a promising candidate for a crop improvement program for generating climate-resilient, disease-resistant cereal crops.

## Supplementary Material

Supplementary material Supplementary file.docx

## Data Availability

The data supporting the findings of this study are included in the Manuscript.
