# Supplementary material for: PgWRKY44-mediated modulation of SA/JA pathways enhances blast pathogen resistance in pearl millet and rice
Source: Plant Signal Behav. 2026 Apr 4;21(1):2650899. doi: 10.1080/15592324.2026.2650899 (PMC13051619; doi:10.1080/15592324.2026.2650899)
Supplement: Supplementary material — Supplementary file.docx [file KPSB_A_2650899_SM2569.docx]

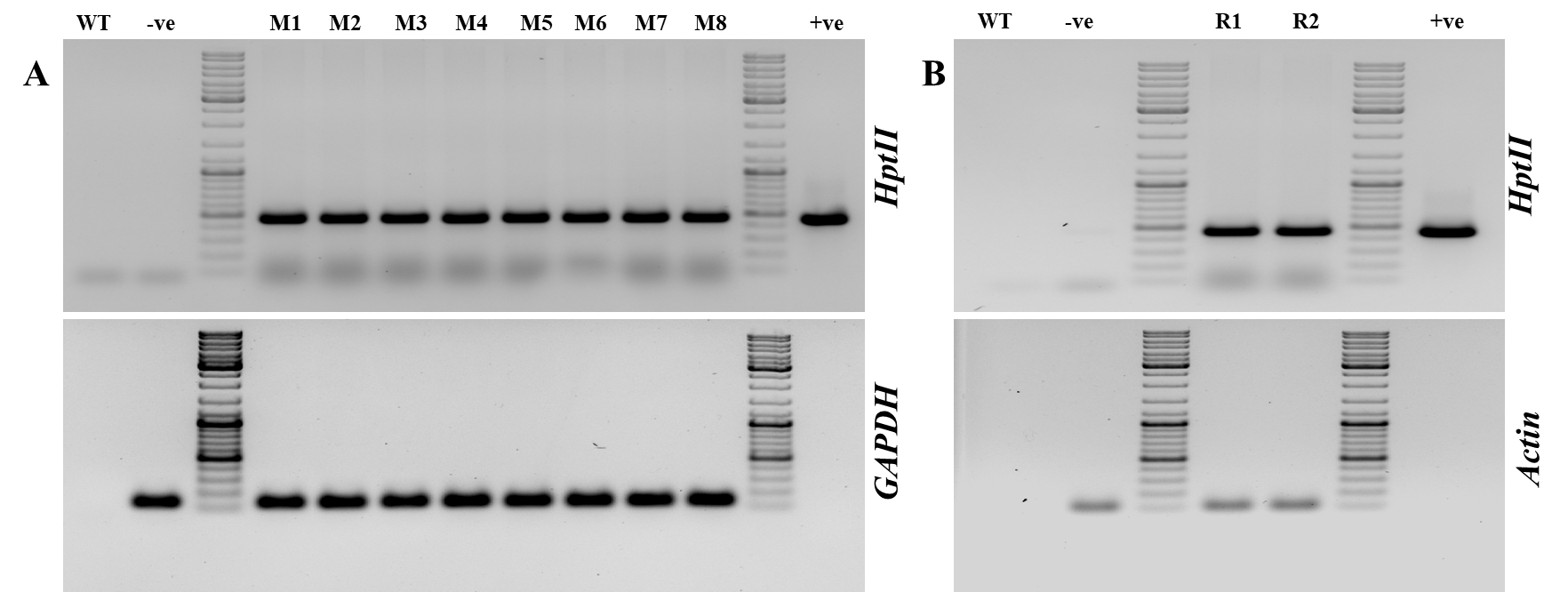


**Supplementary Figure S1: Gene integration analysis using PCR in transgenic PgWRKY44 pearl millet and rice plants.** Electrophoresis of PCR product with specific primer pairs; upper panel: (A) amplification of *HptII* from pearl millet and (B) rice; Lower panel: amplification of housekeeping gene from GAPDH pearl millet (A) and β-Actin from rice (B).WT: Wild type, -ve: negative control, +ve: positive control.


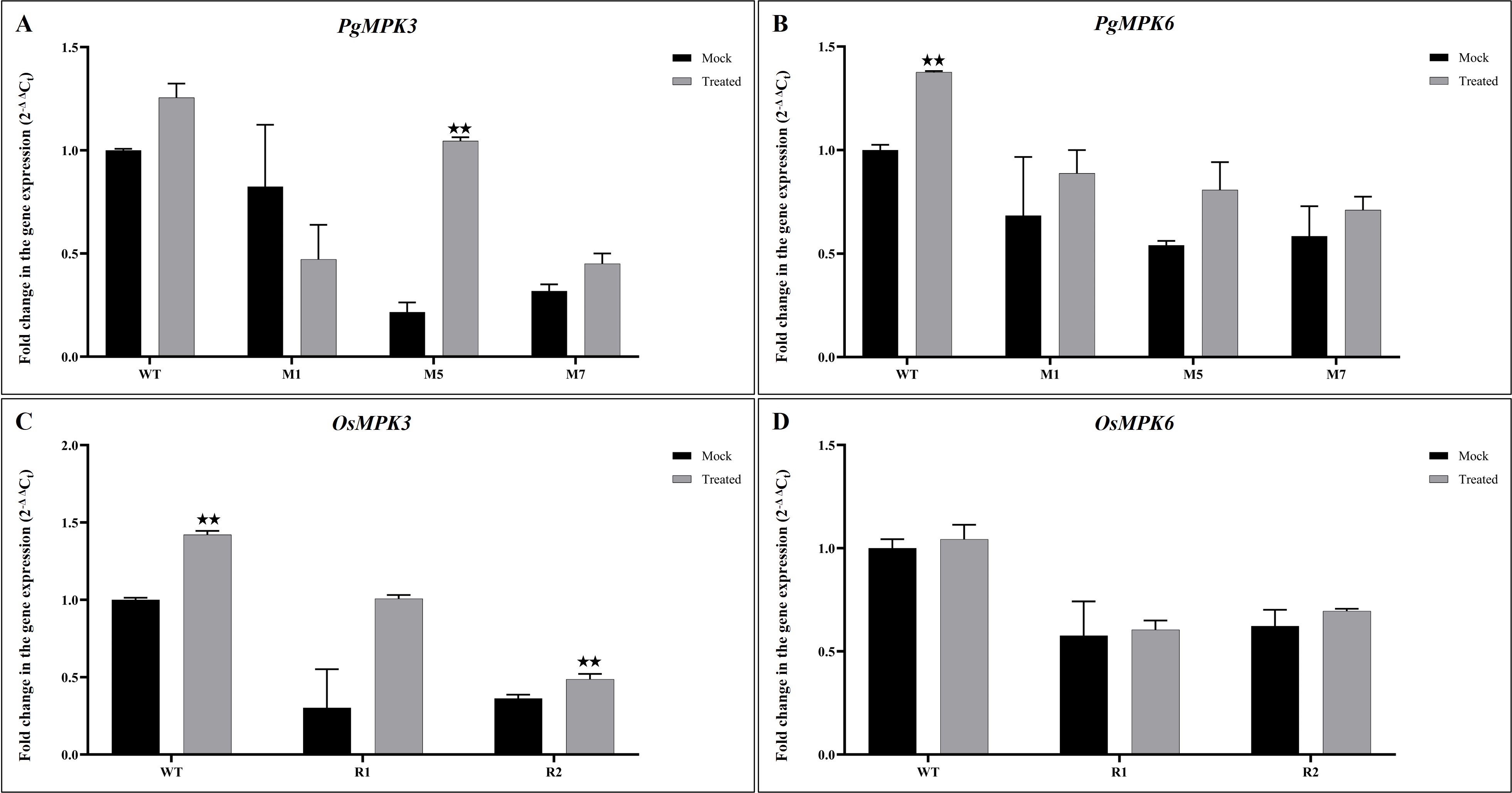


**Supplementary Figure S2: Relative expression profiling of MAP kinases upon pathogen challenge (*M. grisea*).** Relative transcript levels of *MPK3* and *MPK6* were analyzed in wild-type and transgenic lines following pathogen challenge. (A, B) Expression profiles of *PgMPK3* and *PgMPK6* in pearl millet lines (WT, M1, M5, and M7); (C, D) Expression profiles of *OsMPK3* and *OsMPK6* in rice lines (WT, R1, and R2). Data of the three transgenic lines are presented as means (± SDs) of duplicates. Asterisks show significant difference determined using one-way ANOVA compared to control (***p < 0.001; **p < 0.01; *p < 0.05).

**Supplementary table. S1: List of primers used in this study**

| **LIST OF PRIMERS USED IN THIS STUDY** | | | |
| --- | --- | --- | --- |
| **Primer Pair** | | **Purpose** | **Primer sequence (5'-3')** |
| *PgWRKY44* | Forward | Transgenic development | GGATCCATGGAGGAGGTGGAGATGGCCAACAG |
|  | Reverse |  | CCCGGGCTAGGTCTGGGCAGACTGAGCGGG |
| *HptII* | Forward | Transgenic screening | GATGTTGGCGACCTCGTATT |
|  | Reverse |  | TTTAGCGAGAGCCTGACCTATT |
| pCambia T-DNA | Forward | Amplification of gene cassette with hygromycin | ACTCCAATTCACTGTTCCTTGC |
|  | Reverse |  | CTATAGCAGCGGAGGGGTTG |
| *PgGAPDH* | Forward | qRT PCR analysis | TGCCTTGCTCCCCTTGCTAA |
|  | Reverse |  | CAGCCCTTCCACCTCTCCAG |
| *PgWRKY44* | Forward |  | GCGGAAGTATGGGCAAAAG |
|  | Reverse |  | GCCTTCATATGTCACGATTAGC |
| *PgPR1* | Forward |  | TGGACGTGCCGCTGCCG |
|  | Reverse |  | GAACTGCGCCGCCACACG |
| *PgPR10* | Forward |  | GGTTTTCCTCTCCGGCG |
|  | Reverse |  | TCTTCAACACCTTCGCGTC |
| *PgMAPK3* | Forward |  | ATGCTCACCTTCAACCCG |
|  | Reverse |  | ATGTCGTGTAATCGCTCCAG |
| *PgMAPK6* | Forward |  | GGTCTGTGGGCTGTATATTTATGG |
|  | Reverse |  | TTTACGAAGTCCAGATCAGCC |
| *PgCAT* | Forward |  | CAGGAGAGGTTCGTCAAGAG |
|  | Reverse |  | CATCTTCATCCCCAGCGAC |
| *PgPOD* | Forward |  | CTCAACAGAGAGTGCGGTG |
|  | Reverse |  | TCGCGAAGTTGGGGCTGTC |
| *PgSOD* | Forward |  | CCGTATCACTGGACTTGCTC |
|  | Reverse |  | GTTTGGGTTGAAATGTGGTCC |
| *OsActin* | Forward |  | TGTATGCCAGTGGTCGTACC |
|  | Reverse |  | CCAGCAAGGTCGAGACGAA |
| *OsPR1α* | Forward |  | CGTCTTCATCACCTGCAACTACTC |
|  | Reverse |  | CATGCATAACGACGTAGCATAGCA |
| *OsPR10* | Forward |  | CCCTGCCGAATACGCCTAA |
|  | Reverse |  | CTCAAACGCCACGAGAATTTG |
| *OsMAPK3* | Forward |  | CTCATGGATACTGACCTGCATC |
|  | Reverse |  | ATTTCCAGGTTTCAGGTCTCTG |
| *OsMAPK6* | Forward |  | AGAACGGCGAGGAAGTTG |
|  | Reverse |  | GGCGAATTATGTCCTTTATGGC |
| *OsCAT* | Forward |  | GGACGAGGAGGTGGACTACT |
|  | Reverse |  | TGCTTGTGTATCGTCGCCTT |
| *OsPOD* | Forward |  | ACTTCCACGACTGCTTCGTC |
|  | Reverse |  | GACTTGCGGAGGGTCTTGTT |
| *OsSOD* | Forward |  | CTTGGACGGCCAGGATTCAT |
|  | Reverse |  | CTTGGACGGCCAGGATTCAT |
| *OsLOX2* | Forward |  | GCATCCCCAACAGCACATC |
|  | Reverse |  | AATAAAGATTTGGGAGTGACATATTGG |
| *OsAOS2* | Forward |  | CAATACGTGTACTGGTCGAATGG |
|  | Reverse |  | AAGGTGTCGTACCGGAGGAA |
| *OsCHS* | Forward |  | CCGGCGAACTGCGTGTAC |
|  | Reverse |  | TTCCTGATCTGCGACTTGTCA |
| *OsICS1* | Forward |  | TATGGTGCTATCCGCTTCGAT |
|  | Reverse |  | CGAGAACCGAGCTCTCTTCAA |
| *OsPAD4* | Forward |  | GCCAGCTCCCCTACGACTTC |
|  | Reverse |  | CGTGTGCGGTGTAGGTTGTT |
| *OsNH1* | Forward |  | CACGCCTAAGCCTCGGATTA |
|  | Reverse |  | TCAGTGAGCAGCATCCTGACTAG |

**Supplementary table S2: Probable downstream interaction partner of PgWRKY44 identified with STRING online server.**

| **Organism** | **Name of the protein** | **IDENTIFIER** | **Accession No** | **Homologues in the pearl millet genome** |
| --- | --- | --- | --- | --- |
| Arabidopsis | Bifunctional dihydrocamalexate synthase/camalexin synthase | Q9LW27 | AT3G26830.1 | Pgl_GLEAN_10027587 |
|  | Mitogen-activated protein kinase 3 | Q39023 | AT3G45640.1 | Pgl_GLEAN_10025422 |
|  | Sigma factor binding protein 1 | Q9LDH1 | AT3G56710.1 | Pgl_GLEAN_10035731 |
|  | Mitogen-activated protein kinase 6 | Q39026 | AT2G43790.1 | Pgl_GLEAN_10009124 |
|  | Mitogen-activated protein kinase 4 | Q39024 | AT4G01370.1 | Pgl_GLEAN_10023922 |
|  | Sigma factor binding protein 2 | O80669 | AT2G41180.1 | Pgl_GLEAN_10037243 |
| Tobacco | G-box-binding factor 1-like isoform X1. | A0A1S3XXJ9 | LOC107769704 | Pgl_GLEAN_10005031 |
